# Supplementary material for: On growth and form of irregular coiled-shell of a terrestrial snail: Plectostoma concinnum (Fulton, 1901) (Mollusca: Caenogastropoda: Diplommatinidae)
Source: PeerJ. 2014 May 15;2:e383. doi: 10.7717/peerj.383 (PMC4034611; doi:10.7717/peerj.383)

**sults**

Climatic influences

We found no significant climatic differences between the localities (fig. 6 and 7). All localities shared a relative humidity of 95-100% with a minimum humidity of 88% during dry periods. Temperature fluctuated between 23.5 °C and 27.5 °C in shady areas and between 23.5 °C and 28.5 °C in open places. All individuals were found in shaded areas of the rock. During the first week of fieldwork no rain fell, which was visible in slightly higher temperatures and lower relative humidity. This was different for the second period of fieldwork, when it rained nearly every d(appendix).

Temperature and relative humidity for Pangi 1 and Pangi 2.


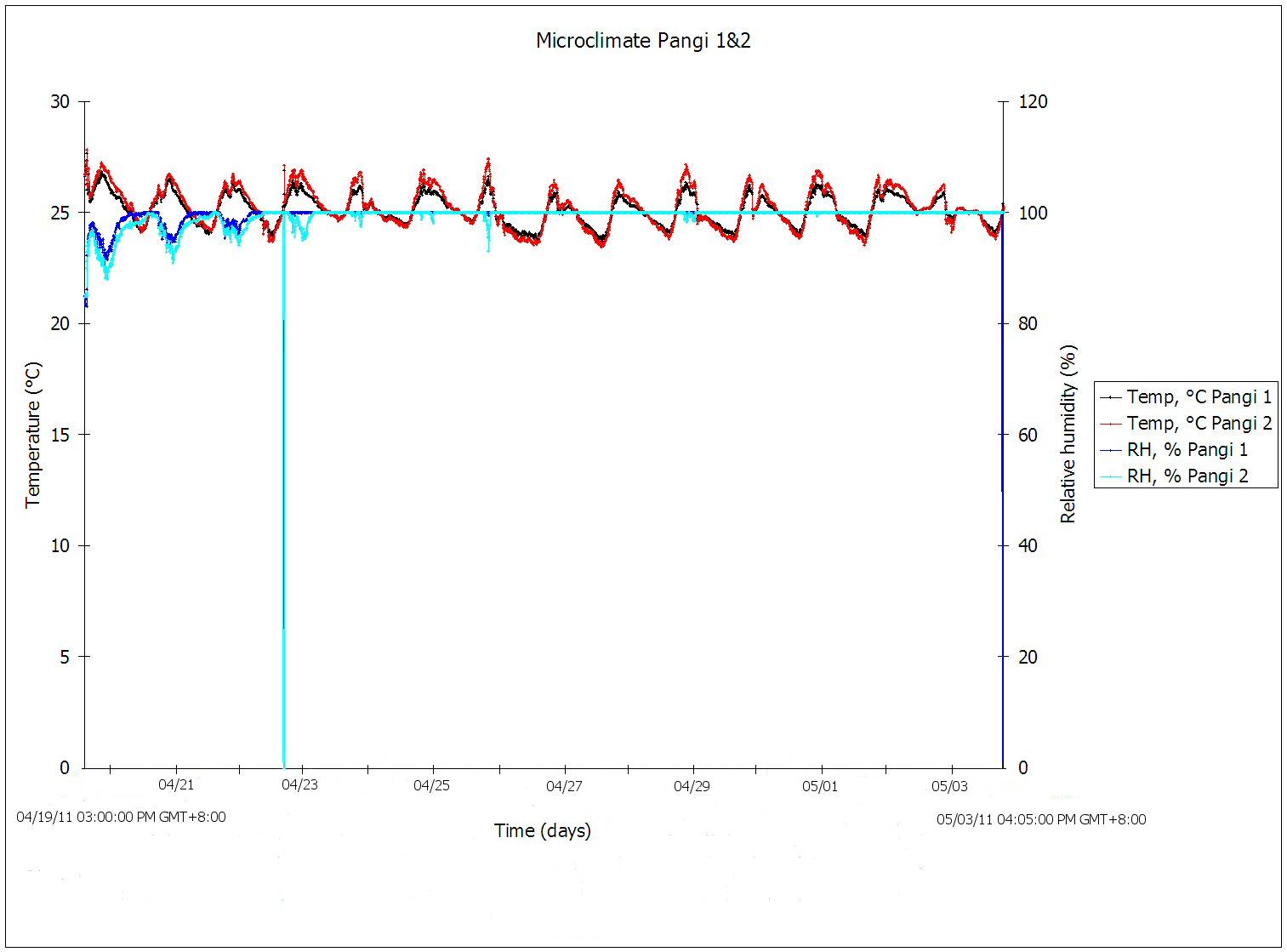

Supplement: File S1 [file peerj-02-383-s001.doc]
