# Supplementary material for: On growth and form of irregular coiled-shell of a terrestrial snail: Plectostoma concinnum (Fulton, 1901) (Mollusca: Caenogastropoda: Diplommatinidae)
Source: PeerJ. 2014 May 15;2:e383. doi: 10.7717/peerj.383 (PMC4034611; doi:10.7717/peerj.383)
Supplement: File S3 [file peerj-02-383-s003.docx]

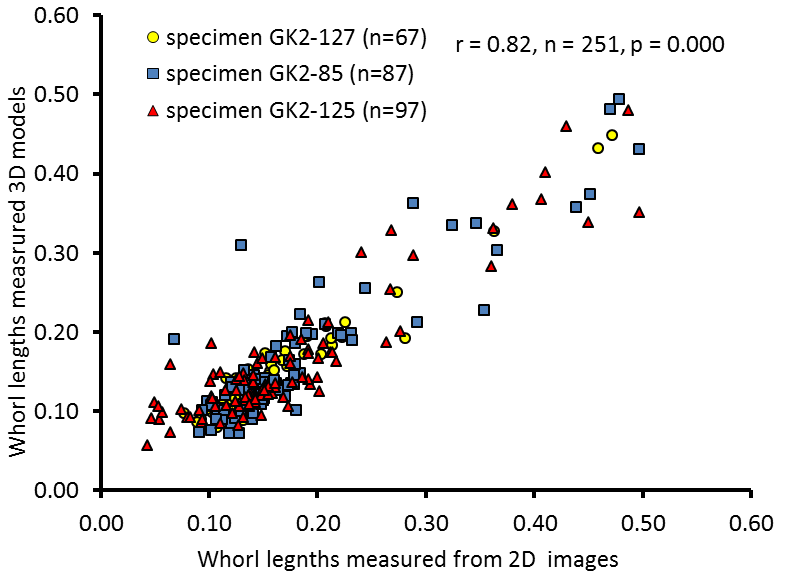


| **Specimen_gap between ribs*** | **2D arc length measurement** | **3D arc length measurement** |
| --- | --- | --- |
| 127_9 | 0.09 | 0.09 |
| 127_10 | 0.09 | 0.09 |
| 127_11 | 0.12 | 0.11 |
| 127_12 | 0.12 | 0.09 |
| 127_13 | 0.12 | 0.10 |
| 127_14 | 0.10 | 0.10 |
| 127_15 | 0.10 | 0.10 |
| 127_16 | 0.13 | 0.10 |
| 127_17 | 0.13 | 0.11 |
| 127_18 | 0.11 | 0.08 |
| 127_19 | 0.11 | 0.09 |
| 127_20 | 0.12 | 0.10 |
| 127_21 | 0.11 | 0.11 |
| 127_22 | 0.13 | 0.11 |
| 127_23 | 0.13 | 0.12 |
| 127_24 | 0.11 | 0.09 |
| 127_25 | 0.13 | 0.11 |
| 127_26 | 0.09 | 0.09 |
| 127_27 | 0.12 | 0.10 |
| 127_28 | 0.11 | 0.10 |
| 127_29 | 0.11 | 0.11 |
| 127_30 | 0.12 | 0.11 |
| 127_31 | 0.08 | 0.10 |
| 127_32 | 0.13 | 0.09 |
| 127_33 | 0.12 | 0.11 |
| 127_34 | 0.12 | 0.10 |
| 127_35 | 0.15 | 0.13 |
| 127_36 | 0.12 | 0.12 |
| 127_37 | 0.13 | 0.12 |
| 127_38 | 0.11 | 0.11 |
| 127_39 | 0.12 | 0.14 |
| 127_40 | 0.14 | 0.15 |
| 127_41 | 0.13 | 0.12 |
| 127_42 | 0.13 | 0.13 |
| 127_43 | 0.15 | 0.14 |
| 127_44 | 0.16 | 0.15 |
| 127_45 | 0.17 | 0.16 |
| 127_46 | 0.16 | 0.15 |
| 127_47 | 0.12 | 0.12 |
| 127_50 | 0.19 | 0.17 |
| 127_51 | 0.15 | 0.12 |
| 127_52 | 0.16 | 0.16 |
| 127_53 | 0.18 | 0.15 |
| 127_54 | 0.21 | 0.18 |
| 127_55 | 0.21 | 0.19 |
| 127_56 | 0.17 | 0.18 |
| 127_57 | 0.21 | 0.21 |
| 127_58 | 0.15 | 0.17 |
| 127_59 | 0.21 | 0.21 |
| 127_60 | 0.22 | 0.19 |
| 127_61 | 0.20 | 0.17 |
| 127_62 | 0.28 | 0.19 |
| 127_63 | 0.17 | 0.16 |
| 127_64 | 0.19 | 0.19 |
| 127_65 | 0.13 | 0.14 |
| 127_66 | 0.13 | 0.13 |
| 127_67 | 0.15 | 0.13 |
| 127_68 | 0.13 | 0.10 |
| 127_69 | 0.13 | 0.13 |
| 127_70 | 0.15 | 0.13 |
| 127_71 | 0.16 | 0.15 |
| 127_72 | 0.15 | 0.13 |
| 127_75 | 0.23 | 0.21 |
| 127_76 | 0.27 | 0.25 |
| 127_77 | 0.36 | 0.33 |
| 127_78 | 0.47 | 0.45 |
| 127_79 | 0.46 | 0.43 |
| 85_4 | 0.09 | 0.07 |
| 85_5 | 0.10 | 0.08 |
| 85_6 | 0.13 | 0.09 |
| 85_7 | 0.11 | 0.10 |
| 85_8 | 0.10 | 0.11 |
| 85_9 | 0.12 | 0.08 |
| 85_10 | 0.10 | 0.11 |
| 85_11 | 0.10 | 0.11 |
| 85_12 | 0.13 | 0.07 |
| 85_13 | 0.10 | 0.11 |
| 85_14 | 0.11 | 0.09 |
| 85_15 | 0.09 | 0.10 |
| 85_16 | 0.12 | 0.09 |
| 85_17 | 0.11 | 0.09 |
| 85_18 | 0.11 | 0.10 |
| 85_19 | 0.11 | 0.09 |
| 85_20 | 0.14 | 0.09 |
| 85_21 | 0.12 | 0.14 |
| 85_22 | 0.13 | 0.10 |
| 85_23 | 0.13 | 0.10 |
| 85_24 | 0.14 | 0.11 |
| 85_25 | 0.14 | 0.14 |
| 85_26 | 0.15 | 0.14 |
| 85_27 | 0.15 | 0.13 |
| 85_28 | 0.13 | 0.11 |
| 85_29 | 0.14 | 0.10 |
| 85_30 | 0.12 | 0.12 |
| 85_31 | 0.14 | 0.11 |
| 85_32 | 0.15 | 0.14 |
| 85_33 | 0.14 | 0.12 |
| 85_34 | 0.13 | 0.13 |
| 85_35 | 0.15 | 0.12 |
| 85_36 | 0.14 | 0.13 |
| 85_37 | 0.15 | 0.13 |
| 85_38 | 0.18 | 0.14 |
| 85_39 | 0.16 | 0.17 |
| 85_40 | 0.15 | 0.11 |
| 85_41 | 0.15 | 0.11 |
| 85_42 | 0.15 | 0.14 |
| 85_43 | 0.16 | 0.14 |
| 85_44 | 0.15 | 0.12 |
| 85_45 | 0.17 | 0.12 |
| 85_46 | 0.16 | 0.14 |
| 85_47 | 0.18 | 0.13 |
| 85_48 | 0.18 | 0.15 |
| 85_49 | 0.17 | 0.19 |
| 85_50 | 0.16 | 0.18 |
| 85_51 | 0.14 | 0.14 |
| 85_52 | 0.18 | 0.19 |
| 85_53 | 0.21 | 0.21 |
| 85_55 | 0.20 | 0.20 |
| 85_56 | 0.17 | 0.16 |
| 85_57 | 0.22 | 0.20 |
| 85_58 | 0.18 | 0.20 |
| 85_59 | 0.23 | 0.20 |
| 85_60 | 0.22 | 0.20 |
| 85_61 | 0.18 | 0.22 |
| 85_62 | 0.19 | 0.20 |
| 85_63 | 0.18 | 0.15 |
| 85_64 | 0.18 | 0.16 |
| 85_65 | 0.13 | 0.15 |
| 85_66 | 0.15 | 0.14 |
| 85_67 | 0.14 | 0.14 |
| 85_68 | 0.12 | 0.13 |
| 85_69 | 0.14 | 0.14 |
| 85_70 | 0.13 | 0.11 |
| 85_71 | 0.16 | 0.13 |
| 85_72 | 0.14 | 0.13 |
| 85_73 | 0.17 | 0.13 |
| 85_74 | 0.23 | 0.19 |
| 85_75 | 0.24 | 0.26 |
| 85_76 | 0.20 | 0.26 |
| 85_77 | 0.35 | 0.23 |
| 85_78 | 0.45 | 0.37 |
| 85_79 | 0.50 | 0.43 |
| 85_80 | 0.48 | 0.49 |
| 85_81 | 0.47 | 0.48 |
| 85_82 | 0.29 | 0.36 |
| 85_83 | 0.13 | 0.31 |
| 85_84 | 0.07 | 0.19 |
| 85_85 | 0.12 | 0.07 |
| 85_86 | 0.18 | 0.10 |
| 85_87 | 0.29 | 0.21 |
| 85_88 | 0.33 | 0.33 |
| 85_89 | 0.37 | 0.30 |
| 85_90 | 0.35 | 0.34 |
| 85_91 | 0.44 | 0.36 |
| 125_7 | 0.13 | 0.09 |
| 125_8 | 0.05 | 0.09 |
| 125_9 | 0.05 | 0.09 |
| 125_10 | 0.05 | 0.11 |
| 125_11 | 0.09 | 0.10 |
| 125_12 | 0.08 | 0.10 |
| 125_13 | 0.13 | 0.15 |
| 125_14 | 0.13 | 0.08 |
| 125_15 | 0.11 | 0.11 |
| 125_16 | 0.08 | 0.09 |
| 125_17 | 0.06 | 0.10 |
| 125_18 | 0.11 | 0.08 |
| 125_19 | 0.12 | 0.10 |
| 125_20 | 0.15 | 0.10 |
| 125_21 | 0.10 | 0.12 |
| 125_22 | 0.14 | 0.11 |
| 125_23 | 0.10 | 0.12 |
| 125_24 | 0.13 | 0.11 |
| 125_25 | 0.08 | 0.09 |
| 125_26 | 0.05 | 0.11 |
| 125_27 | 0.12 | 0.11 |
| 125_28 | 0.14 | 0.12 |
| 125_29 | 0.12 | 0.13 |
| 125_30 | 0.12 | 0.11 |
| 125_31 | 0.14 | 0.12 |
| 125_32 | 0.17 | 0.11 |
| 125_33 | 0.15 | 0.13 |
| 125_34 | 0.14 | 0.12 |
| 125_35 | 0.13 | 0.14 |
| 125_36 | 0.14 | 0.13 |
| 125_37 | 0.13 | 0.11 |
| 125_38 | 0.17 | 0.12 |
| 125_39 | 0.16 | 0.12 |
| 125_40 | 0.15 | 0.13 |
| 125_41 | 0.13 | 0.13 |
| 125_42 | 0.16 | 0.13 |
| 125_43 | 0.14 | 0.11 |
| 125_44 | 0.13 | 0.12 |
| 125_45 | 0.15 | 0.13 |
| 125_46 | 0.15 | 0.12 |
| 125_47 | 0.14 | 0.11 |
| 125_48 | 0.16 | 0.12 |
| 125_49 | 0.16 | 0.13 |
| 125_50 | 0.18 | 0.14 |
| 125_51 | 0.20 | 0.13 |
| 125_52 | 0.15 | 0.13 |
| 125_53 | 0.16 | 0.13 |
| 125_54 | 0.10 | 0.15 |
| 125_55 | 0.14 | 0.14 |
| 125_56 | 0.19 | 0.18 |
| 125_57 | 0.20 | 0.17 |
| 125_58 | 0.18 | 0.17 |
| 125_59 | 0.16 | 0.17 |
| 125_60 | 0.18 | 0.16 |
| 125_61 | 0.19 | 0.13 |
| 125_62 | 0.21 | 0.17 |
| 125_63 | 0.19 | 0.17 |
| 125_64 | 0.18 | 0.20 |
| 125_65 | 0.22 | 0.16 |
| 125_66 | 0.21 | 0.19 |
| 125_67 | 0.28 | 0.20 |
| 125_68 | 0.19 | 0.19 |
| 125_69 | 0.15 | 0.16 |
| 125_70 | 0.10 | 0.18 |
| 125_71 | 0.15 | 0.17 |
| 125_72 | 0.19 | 0.14 |
| 125_73 | 0.11 | 0.15 |
| 125_74 | 0.06 | 0.16 |
| 125_75 | 0.13 | 0.14 |
| 125_76 | 0.14 | 0.14 |
| 125_77 | 0.10 | 0.14 |
| 125_78 | 0.14 | 0.11 |
| 125_79 | 0.14 | 0.17 |
| 125_80 | 0.13 | 0.14 |
| 125_81 | 0.19 | 0.14 |
| 125_82 | 0.20 | 0.14 |
| 125_83 | 0.21 | 0.17 |
| 125_84 | 0.26 | 0.19 |
| 125_85 | 0.27 | 0.25 |
| 125_86 | 0.36 | 0.28 |
| 125_87 | 0.36 | 0.33 |
| 125_88 | 0.50 | 0.35 |
| 125_89 | 0.49 | 0.48 |
| 125_90 | 0.43 | 0.46 |
| 125_91 | 0.41 | 0.40 |
| 125_92 | 0.41 | 0.37 |
| 125_93 | 0.21 | 0.21 |
| 125_94 | 0.04 | 0.06 |
| 125_95 | 0.09 | 0.09 |
| 125_96 | 0.27 | 0.33 |
| 125_97 | 0.06 | 0.07 |
| 125_98 | 0.38 | 0.36 |
| 125_99 | 0.29 | 0.30 |
| 125_100 | 0.19 | 0.21 |
| 125_101 | 0.09 | 0.09 |
| 125_103 | 0.24 | 0.30 |
| 125_104 | 0.45 | 0.34 |

***some 3D arc length cannot be determined from the 3D model because the resolution of the Ct-scan cannot capture the fine ribs (especially those near the protoconch):**

**## specimens_127: ribs no. 1-8, 48-49, 73-74, & 80-82.**

**## specimens_85: ribs no. 1-3, & 54.**

**## specimens_125: ribs no. 1-6, 102.**
