# Supplementary material for: On growth and form of irregular coiled-shell of a terrestrial snail: Plectostoma concinnum (Fulton, 1901) (Mollusca: Caenogastropoda: Diplommatinidae)
Source: PeerJ. 2014 May 15;2:e383. doi: 10.7717/peerj.383 (PMC4034611; doi:10.7717/peerj.383)
Supplement: File S5 [file peerj-02-383-s005.docx]

##A) Checking number of segments and no of points in each segment

curve = bpy.context.active_object.data.splines

No_of_segment = len(bpy.context.active_object.data.splines)

points_in_each_segment = []

for each_segment in list(range(No_of_segment)):

points_of_ith_segment = len(curve[each_segment].bezier_points)

points_in_each_segment.append(points_of_ith_segment)

##B) standardization number of points for each segment

standardized_no_of_points = 200

for each_segment in list(range(No_of_segment)):

while (len(curve[each_segment].bezier_points)) < standardized_no_of_points:

list_of_curvelength=[]

for first_points in ((list(range(len(curve[each_segment].bezier_points))))[:-1]):

the_distance_between_two_points=(((curve[each_segment].bezier_points[(first_points+1)].co)-(curve[each_segment].bezier_points[first_points].co)).length)

list_of_curvelength.append(the_distance_between_two_points)

longest_length_between_points = max(list_of_curvelength)

greatest_gap_index=list_of_curvelength.index(longest_length_between_points)

curve[each_segment].bezier_points[greatest_gap_index].select_control_point=True

bpy.ops.object.mode_set(mode = 'EDIT')

bpy.ops.curve.select_next()

bpy.ops.curve.subdivide()

bpy.ops.curve.select_all()

bpy.ops.object.mode_set(mode = 'OBJECT')

##C) calculate length of each segment and save output

curve = bpy.context.active_object.data.splines

name = bpy.context.active_object.name

No_of_segment = len(bpy.context.active_object.data.splines)

points_in_each_segment = []

for each_segment in list(range(No_of_segment)):

points_of_ith_segment = len(curve[each_segment].bezier_points)

points_in_each_segment.append(points_of_ith_segment)

list_of_length_of_segments = []

for each_spline in list(range(No_of_segment)):

points_from_standardized_segments = len(curve[each_spline].bezier_points)

list_of_length_between_points = []

for begin_point in ((list(range(points_from_standardized_segments)))[:-1]):

the_distance_between_points=(((curve[each_spline].bezier_points[(begin_point+1)].co)-(curve[each_spline].bezier_points[begin_point].co)).length)

list_of_length_between_points.append(the_distance_between_points)

list_of_length_of_segments.append(sum(list_of_length_between_points))

output_temp0 = list_of_length_of_segments.##reverse()

output_temp1 = (str(list_of_length_of_segments)).replace(', ','\n')

output_temp2 = output_temp1.replace('[','')

output = output_temp2.replace(']','')

##Step 12 - write output file in csv format

##Parameters(newfolderpath)

import os

fp=bpy.data.filepath

filepath=os.path.basename(fp)

Blender_file_name=filepath[:-6]

####newfolderpath="c:/E_19072009/Manuscript/PhD thesis/On growth and form of two heteromorphic terrestrial gastropod snails/3D aperture outline analysis/EFA_blender/"

newfolderpath="c:/E_19072009/EFA_blender/"

if not os.path.isdir(newfolderpath):

os.makedirs(newfolderpath)

outputfile1= newfolderpath+" "+Blender_file_name+name+"_analysis"+".csv"

writefile=open(outputfile1, 'w')

writefile.write(output)

writefile.close()

##Script pre-A

##Check broken curve and faciliate broken curve fixing

## Check number of broken segment(s)

import mathutils

CURVE0=bpy.context.active_object.data.splines

CURVE1= CURVE0[0].bezier_points

Number_of_segment = len(CURVE0)

if Number_of_segment >1:

print(Number_of_segment)

CURVE2= CURVE0[1].bezier_points

last_point_of_second_curve=(len(CURVE2))-1

Local_position_of_1st_point_1stCurve= CURVE1[0].select_control_point=True

Local_position_of_last_point_2ndCurve= CURVE2[last_point_of_second_curve].select_control_point=True

##bpy.ops.curve.make_segment()

##A) standardizing number of points for outline

##(Parameter= standard_no_of_point)

##Script i - standardization of number of outline points

CURVE0=()

all_objects=bpy.data.objects

object_ID_name=()

total_number_of_objects=(len(all_objects))

all_object_ID=list(range(total_number_of_objects))

for each_object in all_object_ID:

## CURVE0=all_objects[each_object]

CURVE0=bpy.context.active_object

CURVE1= CURVE0.data.splines[0].bezier_points

points_of_curve=CURVE1

Points_C1=len(CURVE1)

## Output_raw_EFA = ''

## Output_normal_EFA = ''

no_of_points=len(CURVE1)

first_points=0

list_of_curvelength=[]

total_length_between_points=0

standard_no_of_points=800

while first_points <= (no_of_points-2):

the_distance_between_two_points=(((points_of_curve[(first_points+1)].co)-(points_of_curve[first_points].co)).length)

list_of_curvelength.append(the_distance_between_two_points)

total_length_between_points += the_distance_between_two_points

first_points +=1

the_distance_between_1st_lastpoints=(((points_of_curve[0].co)-(points_of_curve[-1].co)).length)

total_length_between_points += the_distance_between_1st_lastpoints

list_of_curvelength.append(the_distance_between_1st_lastpoints)

average_dis=total_length_between_points/standard_no_of_points

greatest_gap=max(list_of_curvelength)

smallest_gap=min(list_of_curvelength)

while greatest_gap > (average_dis * 1.51) and no_of_points <=standard_no_of_points and smallest_gap > 0:

points_of_curve=bpy.context.active_object.data.splines[0].bezier_points

greatest_gap_index=list_of_curvelength.index(greatest_gap)

if greatest_gap_index != ((len(list_of_curvelength))-1):

points_of_curve[greatest_gap_index].select_control_point=True

bpy.ops.object.mode_set(mode = 'EDIT')

bpy.ops.curve.select_next()

no_of_division= round(greatest_gap/(average_dis))

bpy.ops.curve.subdivide(number_cuts=(no_of_division))

bpy.ops.curve.select_all()

bpy.ops.object.mode_set(mode = 'OBJECT')

points_of_curve=bpy.context.active_object.data.splines[0].bezier_points

no_of_points=len(points_of_curve)

first_points=0

list_of_curvelength=[]

total_length_between_points=0

while first_points <= (no_of_points-2):

the_distance_between_two_points=(((points_of_curve[(first_points+1)].co)-(points_of_curve[first_points].co)).length)

list_of_curvelength.append(the_distance_between_two_points)

total_length_between_points += the_distance_between_two_points

first_points +=1

the_distance_between_1st_lastpoints=(((points_of_curve[0].co)-(points_of_curve[-1].co)).length)

total_length_between_points += the_distance_between_1st_lastpoints

list_of_curvelength.append(the_distance_between_1st_lastpoints)

greatest_gap=max(list_of_curvelength)

else:

points_of_curve[0].select_control_point=True

bpy.ops.object.mode_set(mode = 'EDIT')

bpy.ops.curve.select_next()

##no_of_division=round((greatest_gap/average_dis)-1)

bpy.ops.curve.subdivide(number_cuts=1)

bpy.ops.curve.select_all()

bpy.ops.object.mode_set(mode = 'OBJECT')

points_of_curve=bpy.context.active_object.data.splines[0].bezier_points

no_of_points=len(points_of_curve)

first_points=0

list_of_curvelength=[]

total_length_between_points=0

while first_points <= (no_of_points-2):

the_distance_between_two_points=(((points_of_curve[(first_points+1)].co)-(points_of_curve[first_points].co)).length)

list_of_curvelength.append(the_distance_between_two_points)

total_length_between_points += the_distance_between_two_points

first_points +=1

the_distance_between_1st_lastpoints=(((points_of_curve[0].co)-(points_of_curve[-1].co)).length)

total_length_between_points += the_distance_between_1st_lastpoints

list_of_curvelength.append(the_distance_between_1st_lastpoints)

greatest_gap=max(list_of_curvelength)

smallest_gap=min(list_of_curvelength)

greatest_gap=max(list_of_curvelength)

smallest_gap=min(list_of_curvelength)

no_of_points=len(points_of_curve)

while no_of_points > standard_no_of_points:

smallest_gap=min(list_of_curvelength)

smallest_gap_index=list_of_curvelength.index(smallest_gap)

points_of_curve[smallest_gap_index].select_control_point=True

bpy.ops.object.mode_set(mode = 'EDIT')

bpy.ops.curve.delete()

bpy.ops.object.mode_set(mode = 'OBJECT')

points_of_curve=bpy.context.active_object.data.splines[0].bezier_points

no_of_points=len(points_of_curve)

no_of_points

##B) Reorientation: set the homologous landmark as first point

CURVE0=bpy.context.active_object

##Step 4 - Reorientation: set the homologous landmark as first point

##(Parameter= X_reduction_of_point, optional function = to reverse points)

CURVE1=CURVE0.data.splines[0].bezier_points

name=str(CURVE0.name)

Points_C1=len(CURVE1)

list_of_points_length = []

for each_point in list(range(Points_C1)):

point_length = ((CURVE1[each_point].co).length)

list_of_points_length.append(point_length)

position_of_homologous_point = list_of_points_length.index(min(list_of_points_length))

Verts = []

for first_phase_point in list(range(position_of_homologous_point,Points_C1)):#

Verts.append((CURVE1[first_phase_point]).co)#

for second_phase_point in list(range(0,position_of_homologous_point)):#

Verts.append((CURVE1[second_phase_point]).co)#

#list1 = list(range(0,position_of_homologous_point))

#list1.reverse()

#for first_phase_point in list1:

# Verts.append((CURVE1[first_phase_point]).co)

#list2 = list(range(position_of_homologous_point, Points_C1))

#list2.reverse()

#for second_phase_point in list2:

# Verts.append((CURVE1[second_phase_point]).co)

##C) EFA analysis

##Step 7 - calculate EFA analysis from the points of mesh

##(Parameter= no_of_harmonics)

x_coord_mesh = []

y_coord_mesh = []

z_coord_mesh = []

no_of_points = len(Verts)

for each_point in range(no_of_points):

x_coord_mesh.append(Verts[each_point][0])

y_coord_mesh.append(Verts[each_point][1])

z_coord_mesh.append(Verts[each_point][2])

p=len(x_coord_mesh)

no_of_harmonics=5

i=0

Dx_list=[]

Dy_list=[]

Dz_list=[]

Dt_list=[]

firstandlast_x=x_coord_mesh[0]-x_coord_mesh[-1]

Dx_list.append(firstandlast_x)

firstandlast_y=y_coord_mesh[0]-y_coord_mesh[-1]

Dy_list.append(firstandlast_y)

firstandlast_z=z_coord_mesh[0]-z_coord_mesh[-1]

Dz_list.append(firstandlast_z)

firstandlast_t=sqrt(firstandlast_x**2 + firstandlast_y**2 +firstandlast_z**2)

Dt_list.append(firstandlast_t)

while i <= (p-2):

Dx=x_coord_mesh[i+1]-x_coord_mesh[i]

Dx_list.append(Dx)

Dy=y_coord_mesh[i+1]-y_coord_mesh[i]

Dy_list.append(Dy)

Dz=z_coord_mesh[i+1]-z_coord_mesh[i]

Dz_list.append(Dz)

Dt= sqrt(Dx**2 + Dy**2 + Dz**2)

Dt_list.append(Dt)

i +=1

cumsum_i = 0

cumsum_list = []

for each_Dt in Dt_list:

if Dt_list.index((Dt_list[-1])) != -1:

cumsum_i += each_Dt

cumsum_list.append(cumsum_i)

t1 = cumsum_list

t1m1_temp=[float(0)]

t1m1_temp1=t1m1_temp + t1

t1m1=t1m1_temp1[:-1]

T = sum(Dt_list)

temp_output0=[]

list_of_points = list(range(p))

list_of_harmonic = list(range(1,(no_of_harmonics+1)))

harmonics_an=[]

harmonics_bn=[]

harmonics_cn=[]

harmonics_dn=[]

harmonics_en=[]

harmonics_fn=[]

for each_harmonic in list_of_harmonic:

temp_output0=0

for each_point in list_of_points:

a=(Dx_list[each_point]/Dt_list[each_point]) * ((cos (2 * each_harmonic * pi * (t1[each_point]) / T)) -(cos (2 * pi * each_harmonic * (t1m1[each_point]) / T)))

temp_output0 +=a

an = (T/(2 * pi**2 * each_harmonic**2)) * temp_output0

harmonics_an.append(an)

for each_harmonic in list_of_harmonic:

temp_output0=0

for each_point in list_of_points:

b=(Dx_list[each_point]/Dt_list[each_point]) * ((sin (2 * each_harmonic * pi * (t1[each_point]) / T)) -(sin (2 * pi * each_harmonic * (t1m1[each_point]) / T)))

temp_output0 +=b

bn = (T/(2 * pi**2 * each_harmonic**2)) * temp_output0

harmonics_bn.append(bn)

for each_harmonic in list_of_harmonic:

temp_output0=0

for each_point in list_of_points:

c=(Dy_list[each_point]/Dt_list[each_point]) * ((cos (2 * each_harmonic * pi * (t1[each_point]) / T)) -(cos (2 * pi * each_harmonic * (t1m1[each_point]) / T)))

temp_output0 +=c

cn = (T/(2 * pi**2 * each_harmonic**2)) * temp_output0

harmonics_cn.append(cn)

for each_harmonic in list_of_harmonic:

temp_output0=0

for each_point in list_of_points:

d=(Dy_list[each_point]/Dt_list[each_point]) * ((sin (2 * each_harmonic * pi * (t1[each_point]) / T)) -(sin (2 * pi * each_harmonic * (t1m1[each_point]) / T)))

temp_output0 +=d

dn = (T/(2 * pi**2 * each_harmonic**2)) * temp_output0

harmonics_dn.append(dn)

for each_harmonic in list_of_harmonic:

temp_output0=0

for each_point in list_of_points:

e=(Dz_list[each_point]/Dt_list[each_point]) * ((cos (2 * each_harmonic * pi * (t1[each_point]) / T)) -(cos (2 * pi * each_harmonic * (t1m1[each_point]) / T)))

temp_output0 +=e

en = (T/(2 * pi**2 * each_harmonic**2)) * temp_output0

harmonics_en.append(en)

for each_harmonic in list_of_harmonic:

temp_output0=0

for each_point in list_of_points:

f=(Dz_list[each_point]/Dt_list[each_point]) * ((sin (2 * each_harmonic * pi * (t1[each_point]) / T)) -(sin (2 * pi * each_harmonic * (t1m1[each_point]) / T)))

temp_output0 +=f

fn = (T/(2 * pi**2 * each_harmonic**2)) * temp_output0

harmonics_fn.append(fn)

temp_ao=0

for each_point in list_of_points:

temp_output0=(x_coord_mesh[each_point]) * ((Dt_list[each_point])/T)

temp_ao +=temp_output0

ao=2 * temp_ao

temp_co=0

for each_point in list_of_points:

temp_output0=(y_coord_mesh[each_point]) * ((Dt_list[each_point])/T)

temp_co +=temp_output0

co=2 * temp_co

temp_eo=0

for each_point in list_of_points:

temp_output0=(z_coord_mesh[each_point]) * ((Dt_list[each_point])/T)

temp_eo +=temp_output0

eo=2 * temp_eo

harmonics_an

harmonics_bn

harmonics_cn

harmonics_dn

harmonics_en

harmonics_fn

ao

co

eo

combine_output=[harmonics_an, harmonics_bn,harmonics_cn,harmonics_dn,harmonics_en,harmonics_fn,ao,co,eo]

combine_output

##D) Inversion of harmonics from Elliptic fourier Analysis to plot mesh

##Step 9 - Inversion of harmonics from Elliptic fourier Analysis to plot mesh

##(Parameter= n(no_of_vertices), k(no_of_harmonics),)

####Script K - Inversion of harmonics from Elliptic fourier Analysis to plot outline mesh

import math

n=100

k=no_of_harmonics

harmonics_no_index=list(range(1,k+1))

harmonics_value_index=list(range(k))

theta_list_of_points=[]

theta_list_index=list(range(n))

pi_for_each_point=2 * pi / n

for each_point in list(range(n)):

theta_of_each_points = pi_for_each_point * each_point

theta_list_of_points.append(theta_of_each_points)

x_EFA=[]

y_EFA=[]

z_EFA=[]

for each_point_index in theta_list_index:

temp_x_list=[]

for each_harmonics_index in harmonics_value_index:

temp_x=harmonics_an[each_harmonics_index] * cos(harmonics_no_index[each_harmonics_index] * theta_list_of_points[each_point_index]) + harmonics_bn[each_harmonics_index] * sin (harmonics_no_index[each_harmonics_index] * theta_list_of_points[each_point_index])

temp_x_list.append(temp_x)

x_coordEFA=ao/2 + sum(temp_x_list)

x_EFA.append(x_coordEFA)

for each_point_index in theta_list_index:

temp_y_list=[]

for each_harmonics_index in harmonics_value_index:

temp_y=harmonics_cn[each_harmonics_index] * cos(harmonics_no_index[each_harmonics_index] * theta_list_of_points[each_point_index]) + harmonics_dn[each_harmonics_index] * sin (harmonics_no_index[each_harmonics_index] * theta_list_of_points[each_point_index])

temp_y_list.append(temp_y)

y_coordEFA=co/2 + sum(temp_y_list)

y_EFA.append(y_coordEFA)

for each_point_index in theta_list_index:

temp_z_list=[]

for each_harmonics_index in harmonics_value_index:

temp_z=harmonics_en[each_harmonics_index] * cos(harmonics_no_index[each_harmonics_index] * theta_list_of_points[each_point_index]) + harmonics_fn[each_harmonics_index] * sin (harmonics_no_index[each_harmonics_index] * theta_list_of_points[each_point_index])

temp_z_list.append(temp_z)

z_coordEFA=eo/2 + sum(temp_z_list)

z_EFA.append(z_coordEFA)

Verts = []

the_point_index = 0

while the_point_index <= n-1:

unique_vertex=(x_EFA[the_point_index],y_EFA[the_point_index],z_EFA[the_point_index])

Verts.append(unique_vertex)

the_point_index +=1

numberofedges=len(Verts)

list_of_edges=list(range(numberofedges))

Edges=[]

firstedgestart=0

while firstedgestart <= numberofedges-2:

firstpoint=(list_of_edges[firstedgestart])

secondpoint=(list_of_edges[(firstedgestart+1)])

unique_edge=(firstpoint,secondpoint)

Edges.append(unique_edge)

firstedgestart +=1

lastpoint_and_firstpoint=((list_of_edges[-1]),(list_of_edges[0]))

Edges.append(lastpoint_and_firstpoint)

the_temp_mesh=bpy.data.meshes.new((name+'_EFA'))

the_temp_mesh.from_pydata(Verts,Edges,[])

the_temp_mesh.update()

the_th_object=bpy.data.objects.new((name+'_EFA'), the_temp_mesh)

the_th_object.data=the_temp_mesh

scene=bpy.context.scene

scene.objects.link(the_th_object)

the_th_object.select = True

##E) calculate perimeter

all_data = []

total_length_between_points=0

the_distance_between_two_points=0

points_of_curve = bpy.context.active_object.data.vertices

no_of_points = len(points_of_curve)

name = bpy.context.active_object.name

first_points=0

while first_points <= (no_of_points-2):

the_distance_between_two_points=(((points_of_curve[(first_points+1)].co)-(points_of_curve[first_points].co)).length)

total_length_between_points += the_distance_between_two_points

first_points +=1

the_distance_between_1st_lastpoints=(((points_of_curve[0].co)-(points_of_curve[-1].co)).length)

total_length_between_points += the_distance_between_1st_lastpoints

name

total_length_between_points

data = name,total_length_between_points

all_data.append(data)

##F) save and export perimeter data for all outline

Output1 = str(all_data)

Output2 = Output1.replace("), (","\n")

Output3 = Output2.replace("'","")

Output4 = Output3.replace("[(","")

Output = Output4.replace(")]","")

import os

##Step 12 - write output file in csv format

##Parameters(newfolderpath)

fp=bpy.data.filepath

filepath=os.path.basename(fp)

Blender_file_name=filepath[:-6]

####newfolderpath="c:/E_19072009/Manuscript/PhD thesis/On growth and form of two heteromorphic terrestrial gastropod snails/3D aperture outline analysis/EFA_blender/"

newfolderpath="c:/E_19072009/EFA_blender/"

if not os.path.isdir(newfolderpath):

os.makedirs(newfolderpath)

outputfile= newfolderpath+" "+Blender_file_name+" perimetere_new_18082012"+".csv"

writefile=open(outputfile, 'w')

writefile.write(Output)

writefile.close()

##G) normalized EFA analysis

##Step 1 - import module

import bpy

import math

import os

import array

import mathutils

normalised_harmonics_all = []

all_outline = []

Output_raw_EFA = ''

Output_normal_EFA = ''

##Step 8 - normalisation of harmonics from EFA analysis

##(Parameter= k as no_of_harmonics; choose normalization factor: scale, O_inverted, and direction_of_motion )

####Script L - Normalization of harmonics from Elliptic fourier Analysis

k=no_of_harmonics ##see C) EFA analysis

harmonics_value_index=list(range(k))

##Scaling

psi = (1/2) * atan(2 * ((harmonics_an[0] * harmonics_bn[0]) + (harmonics_cn[0] * harmonics_dn[0]) + (harmonics_en[0] * harmonics_fn[0])) / (harmonics_bn[0]**2 + harmonics_dn[0]**2 + harmonics_fn[0]**2 - harmonics_an[0]**2 - harmonics_cn[0]**2 - harmonics_en[0]**2))

a = sqrt (((harmonics_an[0]**2 + harmonics_cn[0]**2 + harmonics_en[0]**2) * cos(psi)**2) + ((harmonics_bn[0]**2 + harmonics_dn[0]**2 + harmonics_fn[0]**2) * sin(psi)**2) - (((harmonics_an[0] * harmonics_bn[0]) + (harmonics_cn[0] * harmonics_dn[0]) + (harmonics_en[0] * harmonics_fn[0])) * sin (2 * psi)))

b = sqrt (((harmonics_an[0]**2 + harmonics_cn[0]**2 + harmonics_en[0]**2) * sin(psi)**2) + ((harmonics_bn[0]**2 + harmonics_dn[0]**2 + harmonics_fn[0]**2) * cos(psi)**2) + (((harmonics_an[0] * harmonics_bn[0]) + (harmonics_cn[0] * harmonics_dn[0]) + (harmonics_en[0] * harmonics_fn[0])) * sin (2 * psi)))

scale = 1/sqrt(pi * a * b)

##Rotation - refered to 1st harmonic

w = a*b/((harmonics_an[0]*harmonics_fn[0]) - (harmonics_bn[0]*harmonics_en[0]))

O21 = ((harmonics_cn[0]*cos(psi)) - (harmonics_dn[0]*sin(psi)))/a

O31 = ((harmonics_en[0]*cos(psi)) - (harmonics_fn[0]*sin(psi)))/a

O22 = ((harmonics_cn[0]*sin(psi)) + (harmonics_dn[0]*cos(psi)))/b

O32 = ((harmonics_en[0]*sin(psi)) + (harmonics_fn[0]*cos(psi)))/b

alpha = ()

if ((harmonics_an[0]*harmonics_fn[0]) - (harmonics_bn[0]*harmonics_en[0])) > 0:

alpha = atan(((harmonics_cn[0]*harmonics_fn[0])-(harmonics_dn[0]*harmonics_en[0]))/((harmonics_an[0]*harmonics_fn[0]) - (harmonics_bn[0]*harmonics_en[0])))

else:

alpha =(atan(((harmonics_cn[0]*harmonics_fn[0])-(harmonics_dn[0]*harmonics_en[0]))/((harmonics_an[0]*harmonics_fn[0]) - (harmonics_bn[0]*harmonics_en[0])))) + pi

beta = acos(w*((O21*O31)+(O22*O32)))

gamma = ()

if O31 > 0:

gamma = acos(O32/sin(beta))

else:

gamma = -acos(O32/sin(beta))

RX_alpha = Matrix (((1,0,0),(0, cos(alpha),-sin(alpha)),(0,sin(alpha),cos(alpha))))

RX_beta = Matrix (((1,0,0),(0, cos(beta),-sin(beta)),(0,sin(beta),cos(beta))))

RX_gamma = Matrix (((1,0,0),(0, cos(gamma),-sin(gamma)),(0,sin(gamma),cos(gamma))))

RY_alpha = Matrix (((cos(alpha),0,sin(alpha)),(0,1,0),(-sin(alpha),0,cos(alpha))))

RY_beta = Matrix (((cos(beta),0,sin(beta)),(0,1,0),(-sin(beta),0,cos(beta))))

RY_gamma = Matrix (((cos(gamma),0,sin(gamma)),(0,1,0),(-sin(gamma),0,cos(gamma))))

RZ_alpha = Matrix (((cos(alpha),-sin(alpha),0),(sin(alpha), cos(alpha),0),(0,0,1)))

RZ_beta = Matrix (((cos(beta),-sin(beta),0),(sin(beta), cos(beta),0),(0,0,1)))

RZ_gamma = Matrix (((cos(gamma),-sin(gamma),0),(sin(gamma), cos(gamma),0),(0,0,1)))

##O=RX_alpha * RY_beta * RZ_gamma ##x1y2z3

##O=RY_alpha * RX_beta * RY_gamma ##y1x2y3

O=RZ_alpha * RX_beta * RZ_gamma ##z1x2z3

##O = Matrix(((((cos(alpha)*cos(gamma)) - (sin(alpha)*cos(beta)*sin(gamma))), ((-cos(alpha)*sin(gamma)) - (sin(alpha)*cos(beta)*cos(gamma))), (sin(alpha)*sin(beta))),(((sin(alpha)*cos(gamma)) + (cos(alpha)*cos(beta)*sin(gamma))), ((-sin(alpha)*sin(gamma)) - (cos(alpha)*cos(beta)*cos(gamma))), (-cos(alpha)*sin(beta))),((sin(beta)*sin(gamma)), (sin(beta)*cos(gamma)),(cos(beta)))))

O_inverted=O.inverted()

direction_of_motion = Matrix(((cos(psi),sin(psi)),((-sin(psi),cos(psi)))))

normalization_factors = scale*O_inverted

##convert each haromonic

normalised_harmonics_an = []

normalised_harmonics_bn = []

normalised_harmonics_cn = []

normalised_harmonics_dn = []

normalised_harmonics_en = []

normalised_harmonics_fn = []

##normalised_harmonics_all = []

for each_harmonic in harmonics_value_index:

the_th_harmonic_matrix = Matrix(((harmonics_an[each_harmonic],harmonics_bn[each_harmonic]),(harmonics_cn[each_harmonic],harmonics_dn[each_harmonic]),(harmonics_en[each_harmonic],harmonics_fn[each_harmonic])))

the_normalised_th_harmonic=normalization_factors*the_th_harmonic_matrix*direction_of_motion

normalised_harmonics_an.append((the_normalised_th_harmonic[0])[0])

normalised_harmonics_bn.append((the_normalised_th_harmonic[0])[1])

normalised_harmonics_cn.append((the_normalised_th_harmonic[1])[0])

normalised_harmonics_dn.append((the_normalised_th_harmonic[1])[1])

normalised_harmonics_en.append((the_normalised_th_harmonic[2])[0])

normalised_harmonics_fn.append((the_normalised_th_harmonic[2])[1])

normalised_harmonics_all.append((the_normalised_th_harmonic[0])[0])

normalised_harmonics_all.append((the_normalised_th_harmonic[0])[1])

normalised_harmonics_all.append((the_normalised_th_harmonic[1])[0])

normalised_harmonics_all.append((the_normalised_th_harmonic[1])[1])

normalised_harmonics_all.append((the_normalised_th_harmonic[2])[0])

normalised_harmonics_all.append((the_normalised_th_harmonic[2])[1])

combine_output_N=[normalised_harmonics_an, normalised_harmonics_bn, normalised_harmonics_cn, normalised_harmonics_dn, normalised_harmonics_en, normalised_harmonics_fn,ao,co,eo]

scale

combine_output_N

all_outline.append(name)

normalised_harmonics_all

all_outline

##H) Inversed Normalized EFA outline

##Step 10 - Inversion of harmonics from Normalised EFA harmonics to plot mesh

##(Parameter= n(no_of_vertices), k(no_of_harmonics),)

####Script M - Inversion of normalized harmonics from Elliptic fourier Analysis to plot outline mesh

n=500

k=no_of_harmonics##see C) EFA analysis & G) normalized EFA

harmonics_no_index=list(range(1,k+1))

harmonics_value_index=list(range(k))

theta_list_of_points=[]

theta_list_index=list(range(n))

pi_for_each_point=2 * pi / n

for each_point in list(range(n)):

theta_of_each_points = pi_for_each_point * each_point

theta_list_of_points.append(theta_of_each_points)

x_NEFA=[]

y_NEFA=[]

z_NEFA=[]

for each_point_index in theta_list_index:

temp_x_list=[]

for each_harmonics_index in harmonics_value_index:

temp_x=normalised_harmonics_an[each_harmonics_index] * cos(harmonics_no_index[each_harmonics_index] * theta_list_of_points[each_point_index]) + normalised_harmonics_bn[each_harmonics_index] * sin (harmonics_no_index[each_harmonics_index] * theta_list_of_points[each_point_index])

temp_x_list.append(temp_x)

x_coordNEFA=ao/2 + sum(temp_x_list)

x_NEFA.append(x_coordNEFA)

for each_point_index in theta_list_index:

temp_y_list=[]

for each_harmonics_index in harmonics_value_index:

temp_y=normalised_harmonics_cn[each_harmonics_index] * cos(harmonics_no_index[each_harmonics_index] * theta_list_of_points[each_point_index]) + normalised_harmonics_dn[each_harmonics_index] * sin (harmonics_no_index[each_harmonics_index] * theta_list_of_points[each_point_index])

temp_y_list.append(temp_y)

y_coordNEFA=co/2 + sum(temp_y_list)

y_NEFA.append(y_coordNEFA)

for each_point_index in theta_list_index:

temp_z_list=[]

for each_harmonics_index in harmonics_value_index:

temp_z=normalised_harmonics_en[each_harmonics_index] * cos(harmonics_no_index[each_harmonics_index] * theta_list_of_points[each_point_index]) + normalised_harmonics_fn[each_harmonics_index] * sin (harmonics_no_index[each_harmonics_index] * theta_list_of_points[each_point_index])

temp_z_list.append(temp_z)

z_coordNEFA=eo/2 + sum(temp_z_list)

z_NEFA.append(z_coordNEFA)

Verts = []

the_point_index = 0

while the_point_index <= n-1:

unique_vertex=(x_NEFA[the_point_index],y_NEFA[the_point_index],z_NEFA[the_point_index])

Verts.append(unique_vertex)

the_point_index +=1

numberofedges=len(Verts)

list_of_edges=list(range(numberofedges))

Edges=[]

firstedgestart=0

while firstedgestart <= numberofedges-2:

firstpoint=(list_of_edges[firstedgestart])

secondpoint=(list_of_edges[(firstedgestart+1)])

unique_edge=(firstpoint,secondpoint)

Edges.append(unique_edge)

firstedgestart +=1

lastpoint_and_firstpoint=((list_of_edges[-1]),(list_of_edges[0]))

Edges.append(lastpoint_and_firstpoint)

the_temp_mesh=bpy.data.meshes.new((name+'_NEFA'))

the_temp_mesh.from_pydata(Verts,Edges,[])

the_temp_mesh.update()

the_th_object=bpy.data.objects.new((name+'_NEFA'), the_temp_mesh)

the_th_object.data=the_temp_mesh

scene=bpy.context.scene

scene.objects.link(the_th_object)

the_th_object.select = True

##I) save output from EFA and Normalized EFA output – C) & G)

##Step 11 - compiling output data from all harmonics from all outlines of each specimen

str_raw_EFA = (((str(combine_output)).replace('[','')).replace(']','')+"\n")

Output_raw_EFA += name + ',' + str_raw_EFA

str_normal_EFA = (((str(combine_output_N)).replace('[','')).replace(']','')+","+str(scale)+"\n")

Output_normal_EFA += name + ',' + str_normal_EFA

normalised_harmonics_all

len(normalised_harmonics_all)

all_outline

##Step 12 - write output file in csv format

##Parameters(newfolderpath)

fp=bpy.data.filepath

filepath=os.path.basename(fp)

Blender_file_name=filepath[:-6]

####newfolderpath="c:/E_19072009/Manuscript/PhD thesis/On growth and form of two heteromorphic terrestrial gastropod snails/3D aperture outline analysis/EFA_blender/"

newfolderpath="c:/E_19072009/EFA_blender/"

if not os.path.isdir(newfolderpath):

os.makedirs(newfolderpath)

outputfile1= newfolderpath+" "+Blender_file_name+" raw_EFA_04112012"+".csv"

outputfile2= newfolderpath+" "+Blender_file_name+" normal_EFA_04112012"+".csv"

writefile=open(outputfile1, 'w')

writefile.write("\n")

writefile.write(Output_raw_EFA)

writefile.close()

writefile=open(outputfile2, 'w')

writefile.write("\n")

writefile.write(Output_normal_EFA)

writefile.close()

##A) Curvature and torsion estimation

import mathutils

q=200

a1=a2=a3=a4=a5=a6=0

bx1=bx2=bx3=by1=by2=by3=bz1=bz2=bz3=0

l_list=[]

l_list_center =[]

s_list=[]

wi_list=[]

list_of_curvature = []

list_of_torsion = []

list_of_binormal_B = []

list_of_normal_N = []

list_of_tangent_T = []

windows_frame=[]

li = 0

si = 0

xi = []

yi = []

zi = []

m = 0

ww=1

points_of_curve=bpy.context.active_object.data.vertices

P=len(points_of_curve)

name = bpy.context.active_object.name

for each_P0 in list(range(q,(P-q))):

windows_frame=[]

windows_frame = list(range((each_P0-q),(each_P0+q+1)))

for each_Pi in windows_frame[:-1]:

si = ((points_of_curve[each_Pi+1].co) - (points_of_curve[each_Pi].co)).length

li += si

l_list.append(li)

s_list.append(si)

for each_Pi in windows_frame[1:]:

xi.append(points_of_curve[each_Pi].co[0])

yi.append(points_of_curve[each_Pi].co[1])

zi.append(points_of_curve[each_Pi].co[2])

m=l_list[q]

for each_li in l_list:

l_list_center.append(each_li-m)

for each_Pi in list(range(q*2)):

wi_list.append((1/(s_list[each_Pi])))##*((l_list_center[each_Pi])**2))

for each_i in list(range(2*q)):

a1 += l_list_center[each_i]**2 * wi_list[each_i]

a2 += (l_list_center[each_i]**3 * wi_list[each_i])/2

a3 += (l_list_center[each_i]**4 * wi_list[each_i])/4

a4 += (l_list_center[each_i]**4 * wi_list[each_i])/6

a5 += (l_list_center[each_i]**5 * wi_list[each_i])/12

a6 += (l_list_center[each_i]**6 * wi_list[each_i])/36

bx1 += wi_list[each_i] * l_list_center[each_i] * (xi[each_i]-points_of_curve[each_P0].co[0])

bx2 += (wi_list[each_i] * (l_list_center[each_i]**2) * (xi[each_i]-points_of_curve[each_P0].co[0]))/2

bx3 += (wi_list[each_i] * (l_list_center[each_i]**3) * (xi[each_i]-points_of_curve[each_P0].co[0]))/6

by1 += wi_list[each_i] * l_list_center[each_i] * (yi[each_i]-points_of_curve[each_P0].co[1])

by2 += (wi_list[each_i] * (l_list_center[each_i]**2) * (yi[each_i]-points_of_curve[each_P0].co[1]))/2

by3 += (wi_list[each_i] * (l_list_center[each_i]**3) * (yi[each_i]-points_of_curve[each_P0].co[1]))/6

bz1 += wi_list[each_i] * l_list_center[each_i] * (zi[each_i]-points_of_curve[each_P0].co[2])

bz2 += (wi_list[each_i] * (l_list_center[each_i]**2) * (zi[each_i]-points_of_curve[each_P0].co[2]))/2

bz3 += (wi_list[each_i] * (l_list_center[each_i]**3) * (zi[each_i]-points_of_curve[each_P0].co[2]))/6

a1_a6_M = Matrix (((a1,a2,a4),(a2,a3,a5),(a4,a5,a6)))

bx_y_z_M = Matrix (((bx1,by1,bz1),(bx2,by2,bz2),(bx3,by3,bz3)))

d_dd_xyz_M = (a1_a6_M.inverted())*bx_y_z_M

Curvature = ((d_dd_xyz_M[0].cross(d_dd_xyz_M[1])).magnitude)/(((d_dd_xyz_M[0]).magnitude)**3)

Torsion = ((d_dd_xyz_M[0].cross(d_dd_xyz_M[1])).dot(d_dd_xyz_M[2]))/ ((((d_dd_xyz_M[0].cross(d_dd_xyz_M[1])).magnitude))**2)

tangent_T = d_dd_xyz_M[0] / d_dd_xyz_M[0].magnitude

normal_N = (d_dd_xyz_M[1] - ((d_dd_xyz_M[1].dot(tangent_T))*tangent_T))/((d_dd_xyz_M[1] - ((d_dd_xyz_M[1].dot(tangent_T))*tangent_T)).magnitude)

binormal_B = tangent_T.cross(normal_N)

Curvature

Torsion

binormal_B

normal_N

tangent_T

list_of_curvature.append(Curvature)

list_of_torsion.append(Torsion)

list_of_binormal_B.append(binormal_B)

list_of_normal_N.append(normal_N)

list_of_tangent_T.append(tangent_T)

a1=a2=a3=a4=a5=a6=0

bx1=bx2=bx3=by1=by2=by3=bz1=bz2=bz3=0

l_list=[]

l_list_center =[]

s_list=[]

wi_list=[]

windows_frame=[]

li = 0

si = 0

xi = []

yi = []

zi = []

m = 0

min(list_of_curvature)

sum(list_of_curvature)/len(list_of_curvature)

max(list_of_curvature)

len(list_of_curvature)

P,q

##B) calculate cumulative length and x,y,z coordinates of spiral

for each_Pi in list(range(P-1)):

xi.append(points_of_curve[each_Pi].co[0])

yi.append(points_of_curve[each_Pi].co[1])

zi.append(points_of_curve[each_Pi].co[2])

si = (((points_of_curve[each_Pi+1].co)-(points_of_curve[each_Pi].co)).length)

s_list.append(si)

list_of_accumulative_arclength = []

si=0

s_list.reverse()

for each_si in s_list:

si += each_si

list_of_accumulative_arclength.append(si)

#list_of_curvature = (list_of_curvature[::q+1])

#list_of_torsion = (list_of_torsion[::q+1])

#list_of_accumulative_arclength = (list_of_accumulative_arclength[q+1::q+1])

##C) prepare output for – curvature, torsion, cumulative length, and x,y,z coordinates of spiral

str_curvature_list = ''

str_torsion_list = ''

Output_torsion = ''

str_acculative_arclength = ''

Output_arclength = ''

Output_curvature_torsion_arclength = ''

str_xi = ''

str_yi = ''

str_zi = ''

str_curvature_list = (((str(list_of_curvature)).replace('[','')).replace(']','')+"\n")

Output_curvature = (',')*q+str_curvature_list

str_torsion_list = (((str(list_of_torsion)).replace('[','')).replace(']','')+"\n")

Output_torsion = (',')*q+str_torsion_list

str_acculative_arclength = (((str(list_of_accumulative_arclength)).replace('[','')).replace(']','')+"\n")

Output_arclength = str_acculative_arclength

str_xi = (str(xi)).replace('[','').replace(']','')+"\n"

Output_x = str_xi

str_yi = (str(yi)).replace('[','').replace(']','')+"\n"

Output_y = str_yi

str_zi = (str(zi)).replace('[','').replace(']','')+"\n"

Output_z = str_zi

Output_curvature_torsion_arclength = Output_curvature+Output_torsion+Output_arclength+Output_x+Output_y+Output_z

##c) save all data – curvature, torsion, cumulative length, and x,y,z coordinates of spiral

import os

fp=bpy.data.filepath

filepath=os.path.basename(fp)

Blender_file_name=filepath[:-6]

####newfolderpath="c:/E_19072009/Manuscript/PhD thesis/On growth and form of two heteromorphic terrestrial gastropod snails/3D aperture outline analysis/EFA_blender/"

newfolderpath="c:/E_19072009/EFA_blender/"

if not os.path.isdir(newfolderpath):

os.makedirs(newfolderpath)

outputfile1= newfolderpath+" "+Blender_file_name+ name + "_q" + (str(q))+".csv"

outputfile2= newfolderpath+" "+Blender_file_name+ name + "torsion_q" + (str(q)) +".csv"

outputfile3= newfolderpath+" "+Blender_file_name+" arclength_w_q" + (str(q)) +".csv"

writefile=open(outputfile1, 'w')

writefile.write("\n")

writefile.write(Output_curvature_torsion_arclength)

writefile.close()

**##Working script 04112012 after standardized number of point**

**##Step 1 of 3 steps**

##B) Reorientation: set the homologous landmark as first point

CURVE0=bpy.context.active_object

##Step 4 - Reorientation: set the homologous landmark as first point

##(Parameter= X_reduction_of_point, optional function = to reverse points)

CURVE1=CURVE0.data.splines[0].bezier_points

name=str(CURVE0.name)

Points_C1=len(CURVE1)

list_of_points_length = []

for each_point in list(range(Points_C1)):

point_length = ((CURVE1[each_point].co).length)

list_of_points_length.append(point_length)

position_of_homologous_point = list_of_points_length.index(min(list_of_points_length))

Verts = []

for first_phase_point in list(range(position_of_homologous_point,Points_C1)):

Verts.append((CURVE1[first_phase_point]).co)

for second_phase_point in list(range(0,position_of_homologous_point)):

Verts.append((CURVE1[second_phase_point]).co)

## reverse orientation

#list1 = list(range(0,position_of_homologous_point))

#list1.reverse()

#for first_phase_point in list1:

# Verts.append((CURVE1[first_phase_point]).co)

#list2 = list(range(position_of_homologous_point, Points_C1))

#list2.reverse()

#for second_phase_point in list2:

# Verts.append((CURVE1[second_phase_point]).co)

**##Step 4 - Reorientation: set the homologous landmark as first point (FOR MESH)**

**##(Parameter= X_reduction_of_point, optional function = to reverse points)**

**Aperture_outline = bpy.context.active_object.data**

**Aperture_outline_vertices = Aperture_outline.vertices**

**name=str(Aperture_outline.name)**

**number_of_vertices= len(Aperture_outline_vertices)**

**list_of_point_length = []**

**for each_vertices in list(range(number_of_vertices)):**

**edge_point_length = ((Aperture_outline_vertices [each_vertices].co).length)**

**list_of_point_length.append(edge_point_length)**

**position_of_homologous_point = list_of_points_length.index(min(list_of_points_length))**

**CURVE1=CURVE0.data.splines[0].bezier_points**

**name=str(CURVE0.name)**

**Points_C1=len(CURVE1)**

**list_of_points_length = []**

**for each_point in list(range(Points_C1)):**

**point_length = ((CURVE1[each_point].co).length)**

**list_of_points_length.append(point_length)**

**position_of_homologous_point = list_of_points_length.index(min(list_of_points_length))**

**Verts = []**

**for first_phase_point in list(range(position_of_homologous_point,Points_C1)):**

**Verts.append((CURVE1[first_phase_point]).co)**

**for second_phase_point in list(range(0,position_of_homologous_point)):**

**Verts.append((CURVE1[second_phase_point]).co)**

**## reverse orientation**

**#list1 = list(range(0,position_of_homologous_point))**

**#list1.reverse()**

**#for first_phase_point in list1:**

**# Verts.append((CURVE1[first_phase_point]).co)**

**#list2 = list(range(position_of_homologous_point, Points_C1))**

**#list2.reverse()**

**#for second_phase_point in list2:**

**# Verts.append((CURVE1[second_phase_point]).co)**

##C) EFA analysis

##Step 7 - calculate EFA analysis from the points of mesh

##(Parameter= no_of_harmonics)

x_coord_mesh = []

y_coord_mesh = []

z_coord_mesh = []

no_of_points = len(Verts)

for each_point in range(no_of_points):

x_coord_mesh.append(Verts[each_point][0])

y_coord_mesh.append(Verts[each_point][1])

z_coord_mesh.append(Verts[each_point][2])

p=len(x_coord_mesh)

no_of_harmonics=5

i=0

Dx_list=[]

Dy_list=[]

Dz_list=[]

Dt_list=[]

firstandlast_x=x_coord_mesh[0]-x_coord_mesh[-1]

Dx_list.append(firstandlast_x)

firstandlast_y=y_coord_mesh[0]-y_coord_mesh[-1]

Dy_list.append(firstandlast_y)

firstandlast_z=z_coord_mesh[0]-z_coord_mesh[-1]

Dz_list.append(firstandlast_z)

firstandlast_t=sqrt(firstandlast_x**2 + firstandlast_y**2 +firstandlast_z**2)

Dt_list.append(firstandlast_t)

while i <= (p-2):

Dx=x_coord_mesh[i+1]-x_coord_mesh[i]

Dx_list.append(Dx)

Dy=y_coord_mesh[i+1]-y_coord_mesh[i]

Dy_list.append(Dy)

Dz=z_coord_mesh[i+1]-z_coord_mesh[i]

Dz_list.append(Dz)

Dt= sqrt(Dx**2 + Dy**2 + Dz**2)

Dt_list.append(Dt)

i +=1

cumsum_i = 0

cumsum_list = []

for each_Dt in Dt_list:

if Dt_list.index((Dt_list[-1])) != -1:

cumsum_i += each_Dt

cumsum_list.append(cumsum_i)

t1 = cumsum_list

t1m1_temp=[float(0)]

t1m1_temp1=t1m1_temp + t1

t1m1=t1m1_temp1[:-1]

T = sum(Dt_list)

temp_output0=[]

list_of_points = list(range(p))

list_of_harmonic = list(range(1,(no_of_harmonics+1)))

harmonics_an=[]

harmonics_bn=[]

harmonics_cn=[]

harmonics_dn=[]

harmonics_en=[]

harmonics_fn=[]

for each_harmonic in list_of_harmonic:

temp_output0=0

for each_point in list_of_points:

a=(Dx_list[each_point]/Dt_list[each_point]) * ((cos (2 * each_harmonic * pi *

(t1[each_point]) / T)) -(cos (2 * pi * each_harmonic * (t1m1[each_point]) / T)))

temp_output0 +=a

an = (T/(2 * pi**2 * each_harmonic**2)) * temp_output0

harmonics_an.append(an)

for each_harmonic in list_of_harmonic:

temp_output0=0

for each_point in list_of_points:

b=(Dx_list[each_point]/Dt_list[each_point]) * ((sin (2 * each_harmonic * pi *

(t1[each_point]) / T)) -(sin (2 * pi * each_harmonic * (t1m1[each_point]) / T)))

temp_output0 +=b

bn = (T/(2 * pi**2 * each_harmonic**2)) * temp_output0

harmonics_bn.append(bn)

for each_harmonic in list_of_harmonic:

temp_output0=0

for each_point in list_of_points:

c=(Dy_list[each_point]/Dt_list[each_point]) * ((cos (2 * each_harmonic * pi *

(t1[each_point]) / T)) -(cos (2 * pi * each_harmonic * (t1m1[each_point]) / T)))

temp_output0 +=c

cn = (T/(2 * pi**2 * each_harmonic**2)) * temp_output0

harmonics_cn.append(cn)

for each_harmonic in list_of_harmonic:

temp_output0=0

for each_point in list_of_points:

d=(Dy_list[each_point]/Dt_list[each_point]) * ((sin (2 * each_harmonic * pi *

(t1[each_point]) / T)) -(sin (2 * pi * each_harmonic * (t1m1[each_point]) / T)))

temp_output0 +=d

dn = (T/(2 * pi**2 * each_harmonic**2)) * temp_output0

harmonics_dn.append(dn)

for each_harmonic in list_of_harmonic:

temp_output0=0

for each_point in list_of_points:

e=(Dz_list[each_point]/Dt_list[each_point]) * ((cos (2 * each_harmonic * pi *

(t1[each_point]) / T)) -(cos (2 * pi * each_harmonic * (t1m1[each_point]) / T)))

temp_output0 +=e

en = (T/(2 * pi**2 * each_harmonic**2)) * temp_output0

harmonics_en.append(en)

for each_harmonic in list_of_harmonic:

temp_output0=0

for each_point in list_of_points:

f=(Dz_list[each_point]/Dt_list[each_point]) * ((sin (2 * each_harmonic * pi *

(t1[each_point]) / T)) -(sin (2 * pi * each_harmonic * (t1m1[each_point]) / T)))

temp_output0 +=f

fn = (T/(2 * pi**2 * each_harmonic**2)) * temp_output0

harmonics_fn.append(fn)

temp_ao=0

for each_point in list_of_points:

temp_output0=(x_coord_mesh[each_point]) * ((Dt_list[each_point])/T)

temp_ao +=temp_output0

ao=2 * temp_ao

temp_co=0

for each_point in list_of_points:

temp_output0=(y_coord_mesh[each_point]) * ((Dt_list[each_point])/T)

temp_co +=temp_output0

co=2 * temp_co

temp_eo=0

for each_point in list_of_points:

temp_output0=(z_coord_mesh[each_point]) * ((Dt_list[each_point])/T)

temp_eo +=temp_output0

eo=2 * temp_eo

harmonics_an

harmonics_bn

harmonics_cn

harmonics_dn

harmonics_en

harmonics_fn

ao

co

eo

combine_output=[harmonics_an,

harmonics_bn,harmonics_cn,harmonics_dn,harmonics_en,harmonics_fn,ao,co,eo]

combine_output

##D) Inversion of harmonics from Elliptic fourier Analysis to plot mesh

##Step 9 - Inversion of harmonics from Elliptic fourier Analysis to plot mesh

##(Parameter= n(no_of_vertices), k(no_of_harmonics),)

####Script K - Inversion of harmonics from Elliptic fourier Analysis to plot outline mesh

import math

n=100

k=no_of_harmonics

harmonics_no_index=list(range(1,k+1))

harmonics_value_index=list(range(k))

theta_list_of_points=[]

theta_list_index=list(range(n))

pi_for_each_point=2 * pi / n

for each_point in list(range(n)):

theta_of_each_points = pi_for_each_point * each_point

theta_list_of_points.append(theta_of_each_points)

x_EFA=[]

y_EFA=[]

z_EFA=[]

for each_point_index in theta_list_index:

temp_x_list=[]

for each_harmonics_index in harmonics_value_index:

temp_x=harmonics_an[each_harmonics_index] * cos(harmonics_no_index[each_harmonics_index] * theta_list_of_points[each_point_index]) + harmonics_bn[each_harmonics_index] * sin (harmonics_no_index[each_harmonics_index] * theta_list_of_points[each_point_index])

temp_x_list.append(temp_x)

x_coordEFA=ao/2 + sum(temp_x_list)

x_EFA.append(x_coordEFA)

for each_point_index in theta_list_index:

temp_y_list=[]

for each_harmonics_index in harmonics_value_index:

temp_y=harmonics_cn[each_harmonics_index] * cos(harmonics_no_index[each_harmonics_index] * theta_list_of_points[each_point_index]) + harmonics_dn[each_harmonics_index] * sin (harmonics_no_index[each_harmonics_index] *theta_list_of_points[each_point_index])

temp_y_list.append(temp_y)

y_coordEFA=co/2 + sum(temp_y_list)

y_EFA.append(y_coordEFA)

for each_point_index in theta_list_index:

temp_z_list=[]

for each_harmonics_index in harmonics_value_index:

temp_z=harmonics_en[each_harmonics_index] * cos(harmonics_no_index[each_harmonics_index] * theta_list_of_points[each_point_index]) + harmonics_fn[each_harmonics_index] * sin (harmonics_no_index[each_harmonics_index] * theta_list_of_points[each_point_index])

temp_z_list.append(temp_z)

z_coordEFA=eo/2 + sum(temp_z_list)

z_EFA.append(z_coordEFA)

Verts = []

the_point_index = 0

while the_point_index <= n-1:

unique_vertex=(x_EFA[the_point_index],y_EFA[the_point_index],z_EFA[the_point_index])

Verts.append(unique_vertex)

the_point_index +=1

numberofedges=len(Verts)

list_of_edges=list(range(numberofedges))

Edges=[]

firstedgestart=0

while firstedgestart <= numberofedges-2:

firstpoint=(list_of_edges[firstedgestart])

secondpoint=(list_of_edges[(firstedgestart+1)])

unique_edge=(firstpoint,secondpoint)

Edges.append(unique_edge)

firstedgestart +=1

lastpoint_and_firstpoint=((list_of_edges[-1]),(list_of_edges[0]))

Edges.append(lastpoint_and_firstpoint)

the_temp_mesh=bpy.data.meshes.new((name+'_EFA'))

the_temp_mesh.from_pydata(Verts,Edges,[])

the_temp_mesh.update()

the_th_object=bpy.data.objects.new((name+'_EFA'), the_temp_mesh)

the_th_object.data=the_temp_mesh

scene=bpy.context.scene

scene.objects.link(the_th_object)

the_th_object.select = True

**##Step 2 of 3 steps**

##E) calculate perimeter

#all_data = [] #silent this after used for the first time

total_length_between_points=0

the_distance_between_two_points=0

points_of_curve = bpy.context.active_object.data.vertices

no_of_points = len(points_of_curve)

name = bpy.context.active_object.name

first_points=0

while first_points <= (no_of_points-2):

the_distance_between_two_points=(((points_of_curve[(first_points+1)].co)-

(points_of_curve[first_points].co)).length)

total_length_between_points += the_distance_between_two_points

first_points +=1

the_distance_between_1st_lastpoints=(((points_of_curve[0].co)-(points_of_curve[-

1].co)).length)

total_length_between_points += the_distance_between_1st_lastpoints

name

total_length_between_points

data = name,total_length_between_points

all_data.append(data)

##G) normalized EFA analysis

##Step 1 - import module

import bpy

import math

import os

import array

import mathutils

normalised_harmonics_all = []

all_outline = []

#Output_raw_EFA = '' #silent this after used for the first time

#Output_normal_EFA = '' #silent this after used for the first time

##Step 8 - normalisation of harmonics from EFA analysis

##(Parameter= k as no_of_harmonics; choose normalization factor: scale, O_inverted, and direction_of_motion)

####Script L - Normalization of harmonics from Elliptic fourier Analysis

k=no_of_harmonics ##see C) EFA analysis

harmonics_value_index=list(range(k))

##Scaling

psi = (1/2) * atan(2 * ((harmonics_an[0] * harmonics_bn[0]) + (harmonics_cn[0] * harmonics_dn[0]) + (harmonics_en[0] * harmonics_fn[0])) / (harmonics_bn[0]**2 + harmonics_dn[0]**2 + harmonics_fn[0]**2 - harmonics_an[0]**2 - harmonics_cn[0]**2 - harmonics_en[0]**2))

a = sqrt (((harmonics_an[0]**2 + harmonics_cn[0]**2 + harmonics_en[0]**2) * cos(psi)**2) + ((harmonics_bn[0]**2 + harmonics_dn[0]**2 + harmonics_fn[0]**2) * sin(psi)**2) - (((harmonics_an[0] * harmonics_bn[0]) + (harmonics_cn[0] * harmonics_dn[0]) + (harmonics_en[0] * harmonics_fn[0])) * sin (2 * psi)))

b = sqrt (((harmonics_an[0]**2 + harmonics_cn[0]**2 + harmonics_en[0]**2) * sin(psi)**2) + ((harmonics_bn[0]**2 + harmonics_dn[0]**2 + harmonics_fn[0]**2) * cos(psi)**2) + (((harmonics_an[0] * harmonics_bn[0]) + (harmonics_cn[0] * harmonics_dn[0]) + (harmonics_en[0] * harmonics_fn[0])) * sin (2 * psi)))

scale = 1/sqrt(pi * a * b)

##Rotation - refered to 1st harmonic

w = a*b/((harmonics_an[0]*harmonics_fn[0]) - (harmonics_bn[0]*harmonics_en[0]))

O21 = ((harmonics_cn[0]*cos(psi)) - (harmonics_dn[0]*sin(psi)))/a

O31 = ((harmonics_en[0]*cos(psi)) - (harmonics_fn[0]*sin(psi)))/a

O22 = ((harmonics_cn[0]*sin(psi)) + (harmonics_dn[0]*cos(psi)))/b

O32 = ((harmonics_en[0]*sin(psi)) + (harmonics_fn[0]*cos(psi)))/b

alpha = ()

if ((harmonics_an[0]*harmonics_fn[0]) - (harmonics_bn[0]*harmonics_en[0])) > 0:

alpha = atan(((harmonics_cn[0]*harmonics_fn[0])-(harmonics_dn[0]*harmonics_en[0]))/

((harmonics_an[0]*harmonics_fn[0]) - (harmonics_bn[0]*harmonics_en[0])))

else:

alpha =(atan(((harmonics_cn[0]*harmonics_fn[0])-(harmonics_dn[0]*harmonics_en[0]))/((harmonics_an[0]*harmonics_fn[0]) - (harmonics_bn[0]*harmonics_en[0])))) + pi

beta = acos(w*((O21*O31)+(O22*O32)))

gamma = ()

if O31 > 0:

gamma = acos(O32/sin(beta))

else:

gamma = -acos(O32/sin(beta))

RX_alpha = Matrix (((1,0,0),(0, cos(alpha),-sin(alpha)),(0,sin(alpha),cos(alpha))))

RX_beta = Matrix (((1,0,0),(0, cos(beta),-sin(beta)),(0,sin(beta),cos(beta))))

RX_gamma = Matrix (((1,0,0),(0, cos(gamma),-sin(gamma)),(0,sin(gamma),cos(gamma))))

RY_alpha = Matrix (((cos(alpha),0,sin(alpha)),(0,1,0),(-sin(alpha),0,cos(alpha))))

RY_beta = Matrix (((cos(beta),0,sin(beta)),(0,1,0),(-sin(beta),0,cos(beta))))

RY_gamma = Matrix (((cos(gamma),0,sin(gamma)),(0,1,0),(-sin(gamma),0,cos(gamma))))

RZ_alpha = Matrix (((cos(alpha),-sin(alpha),0),(sin(alpha), cos(alpha),0),(0,0,1)))

RZ_beta = Matrix (((cos(beta),-sin(beta),0),(sin(beta), cos(beta),0),(0,0,1)))

RZ_gamma = Matrix (((cos(gamma),-sin(gamma),0),(sin(gamma), cos(gamma),0),(0,0,1)))

##O=RX_alpha * RY_beta * RZ_gamma ##x1y2z3

##O=RY_alpha * RX_beta * RY_gamma ##y1x2y3

O=RZ_alpha * RX_beta * RZ_gamma ##z1x2z3

##O = Matrix(((((cos(alpha)*cos(gamma)) - (sin(alpha)*cos(beta)*sin(gamma))), ((-cos(alpha)*sin(gamma)) - (sin(alpha)*cos(beta)*cos(gamma))), (sin(alpha)*sin(beta))),(((sin(alpha)*cos(gamma)) + (cos(alpha)*cos(beta)*sin(gamma))), ((-sin(alpha)*sin(gamma)) - (cos(alpha)*cos(beta)*cos(gamma))), (-cos(alpha)*sin(beta))),((sin(beta)*sin(gamma)), (sin(beta)*cos(gamma)),(cos(beta)))))

O_inverted=O.inverted()

direction_of_motion = Matrix(((cos(psi),sin(psi)),((-sin(psi),cos(psi)))))

normalization_factors = scale*O_inverted

##convert each haromonic

normalised_harmonics_an = []

normalised_harmonics_bn = []

normalised_harmonics_cn = []

normalised_harmonics_dn = []

normalised_harmonics_en = []

normalised_harmonics_fn = []

##normalised_harmonics_all = []

for each_harmonic in harmonics_value_index:

the_th_harmonic_matrix = Matrix(((harmonics_an[each_harmonic],harmonics_bn[each_harmonic]),(harmonics_cn[each_harmonic],harmonics_dn[each_harmonic]),(harmonics_en[each_harmonic],harmonics_fn[each_harmonic])))

the_normalised_th_harmonic=normalization_factors*the_th_harmonic_matrix*direction_of_motion

normalised_harmonics_an.append((the_normalised_th_harmonic[0])[0])

normalised_harmonics_bn.append((the_normalised_th_harmonic[0])[1])

normalised_harmonics_cn.append((the_normalised_th_harmonic[1])[0])

normalised_harmonics_dn.append((the_normalised_th_harmonic[1])[1])

normalised_harmonics_en.append((the_normalised_th_harmonic[2])[0])

normalised_harmonics_fn.append((the_normalised_th_harmonic[2])[1])

normalised_harmonics_all.append((the_normalised_th_harmonic[0])[0])

normalised_harmonics_all.append((the_normalised_th_harmonic[0])[1])

normalised_harmonics_all.append((the_normalised_th_harmonic[1])[0])

normalised_harmonics_all.append((the_normalised_th_harmonic[1])[1])

normalised_harmonics_all.append((the_normalised_th_harmonic[2])[0])

normalised_harmonics_all.append((the_normalised_th_harmonic[2])[1])

combine_output_N=[normalised_harmonics_an, normalised_harmonics_bn,

normalised_harmonics_cn, normalised_harmonics_dn, normalised_harmonics_en,

normalised_harmonics_fn,ao,co,eo]

scale

combine_output_N

all_outline.append(name)

normalised_harmonics_all

all_outline

##H) Inversed Normalized EFA outline

##Step 10 - Inversion of harmonics from Normalised EFA harmonics to plot mesh

##(Parameter= n(no_of_vertices), k(no_of_harmonics),)

####Script M - Inversion of normalized harmonics from Elliptic fourier Analysis to plot

outline mesh

n=500

k=no_of_harmonics##see C) EFA analysis & G) normalized EFA

harmonics_no_index=list(range(1,k+1))

harmonics_value_index=list(range(k))

theta_list_of_points=[]

theta_list_index=list(range(n))

pi_for_each_point=2 * pi / n

for each_point in list(range(n)):

theta_of_each_points = pi_for_each_point * each_point

theta_list_of_points.append(theta_of_each_points)

x_NEFA=[]

y_NEFA=[]

z_NEFA=[]

for each_point_index in theta_list_index:

temp_x_list=[]

for each_harmonics_index in harmonics_value_index:

temp_x=normalised_harmonics_an[each_harmonics_index] * cos(harmonics_no_index[each_harmonics_index] * theta_list_of_points[each_point_index]) +normalised_harmonics_bn[each_harmonics_index] * sin (harmonics_no_index[each_harmonics_index] * theta_list_of_points[each_point_index])

temp_x_list.append(temp_x)

x_coordNEFA=ao/2 + sum(temp_x_list)

x_NEFA.append(x_coordNEFA)

for each_point_index in theta_list_index:

temp_y_list=[]

for each_harmonics_index in harmonics_value_index:

temp_y=normalised_harmonics_cn[each_harmonics_index] * cos(harmonics_no_index[each_harmonics_index] * theta_list_of_points[each_point_index]) +normalised_harmonics_dn[each_harmonics_index] * sin (harmonics_no_index[each_harmonics_index] * theta_list_of_points[each_point_index])

temp_y_list.append(temp_y)

y_coordNEFA=co/2 + sum(temp_y_list)

y_NEFA.append(y_coordNEFA)

for each_point_index in theta_list_index:

temp_z_list=[]

for each_harmonics_index in harmonics_value_index:

temp_z=normalised_harmonics_en[each_harmonics_index] * cos(harmonics_no_index[each_harmonics_index] * theta_list_of_points[each_point_index]) +normalised_harmonics_fn[each_harmonics_index] * sin (harmonics_no_index[each_harmonics_index] * theta_list_of_points[each_point_index])

temp_z_list.append(temp_z)

z_coordNEFA=eo/2 + sum(temp_z_list)

z_NEFA.append(z_coordNEFA)

Verts = []

the_point_index = 0

while the_point_index <= n-1:

unique_vertex=(x_NEFA[the_point_index],y_NEFA[the_point_index],z_NEFA

[the_point_index])

Verts.append(unique_vertex)

the_point_index +=1

numberofedges=len(Verts)

list_of_edges=list(range(numberofedges))

Edges=[]

firstedgestart=0

while firstedgestart <= numberofedges-2:

firstpoint=(list_of_edges[firstedgestart])

secondpoint=(list_of_edges[(firstedgestart+1)])

unique_edge=(firstpoint,secondpoint)

Edges.append(unique_edge)

firstedgestart +=1

lastpoint_and_firstpoint=((list_of_edges[-1]),(list_of_edges[0]))

Edges.append(lastpoint_and_firstpoint)

the_temp_mesh=bpy.data.meshes.new((name+'_NEFA'))

the_temp_mesh.from_pydata(Verts,Edges,[])

the_temp_mesh.update()

the_th_object=bpy.data.objects.new((name+'_NEFA'), the_temp_mesh)

the_th_object.data=the_temp_mesh

scene=bpy.context.scene

scene.objects.link(the_th_object)

the_th_object.select = True

##I) save output from EFA and Normalized EFA output – C) & G)

##Step 11 - compiling output data from all harmonics from all outlines of each specimen

str_raw_EFA = (((str(combine_output)).replace('[','')).replace(']','')+"\n")

Output_raw_EFA += name + ',' + str_raw_EFA

str_normal_EFA = (((str(combine_output_N)).replace('[','')).replace(']','')+","+str

(scale)+"\n")

Output_normal_EFA += name + ',' + str_normal_EFA

normalised_harmonics_all

len(normalised_harmonics_all)

all_outline

**##Step 3 of 3 steps after finished step 1 and 2 for all outlines**

##F) save and export perimeter data for all outline

Output1 = str(all_data)

Output2 = Output1.replace("), (","\n")

Output3 = Output2.replace("'","")

Output4 = Output3.replace("[(","")

Output = Output4.replace(")]","")

import os

##Step 12 - write output file in csv format

##Parameters(newfolderpath)

fp=bpy.data.filepath

filepath=os.path.basename(fp)

Blender_file_name=filepath[:-6]

####newfolderpath="c:/E_19072009/Manuscript/PhD thesis/On growth and form of two heteromorphic terrestrial gastropod snails/3D aperture outline analysis/EFA_blender/"

newfolderpath="c:/E_19072009/abc/"

if not os.path.isdir(newfolderpath):

os.makedirs(newfolderpath)

outputfile= newfolderpath+" "+Blender_file_name+" abc_new_04112012"+".csv"

writefile=open(outputfile, 'w')

writefile.write(Output)

writefile.close()

##Step 12 - write output file in csv format

##Parameters(newfolderpath)

fp=bpy.data.filepath

filepath=os.path.basename(fp)

Blender_file_name=filepath[:-6]

####newfolderpath="c:/E_19072009/Manuscript/PhD thesis/On growth and form of two heteromorphic terrestrial gastropod snails/3D aperture outline analysis/EFA_blender/"

newfolderpath="c:/E_19072009/abc/"

if not os.path.isdir(newfolderpath):

os.makedirs(newfolderpath)

outputfile1= newfolderpath+" "+Blender_file_name+" raw_EFA_abc04112012"+".csv"

outputfile2= newfolderpath+" "+Blender_file_name+" normal_EFA_abc04112012"+".csv"

writefile=open(outputfile1, 'w')

writefile.write("\n")

writefile.write(Output_raw_EFA)

writefile.close()

writefile=open(outputfile2, 'w')

writefile.write("\n")

writefile.write(Output_normal_EFA)

writefile.close()
