# Supplementary material for: On growth and form of irregular coiled-shell of a terrestrial snail: Plectostoma concinnum (Fulton, 1901) (Mollusca: Caenogastropoda: Diplommatinidae)
Source: PeerJ. 2014 May 15;2:e383. doi: 10.7717/peerj.383 (PMC4034611; doi:10.7717/peerj.383)
Supplement: File S7 [file peerj-02-383-s007.docx]

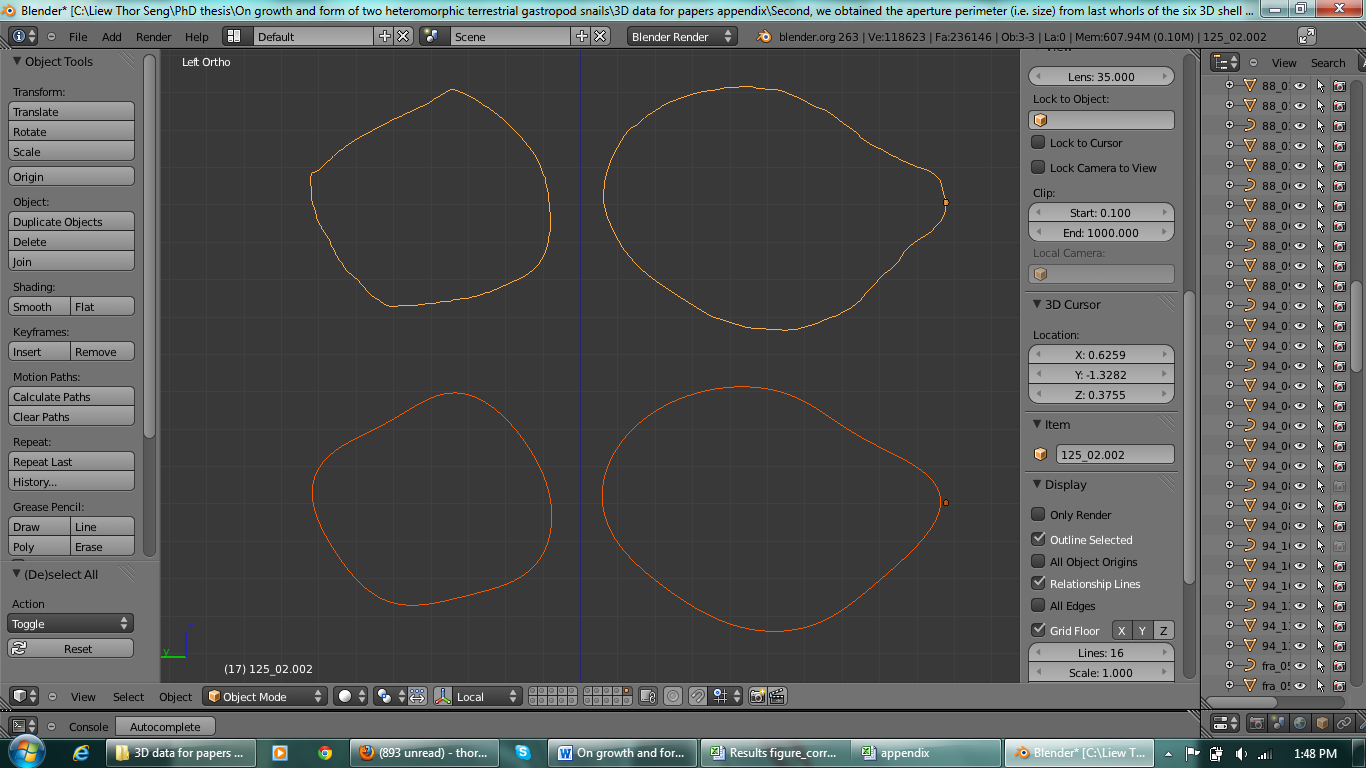


**A**


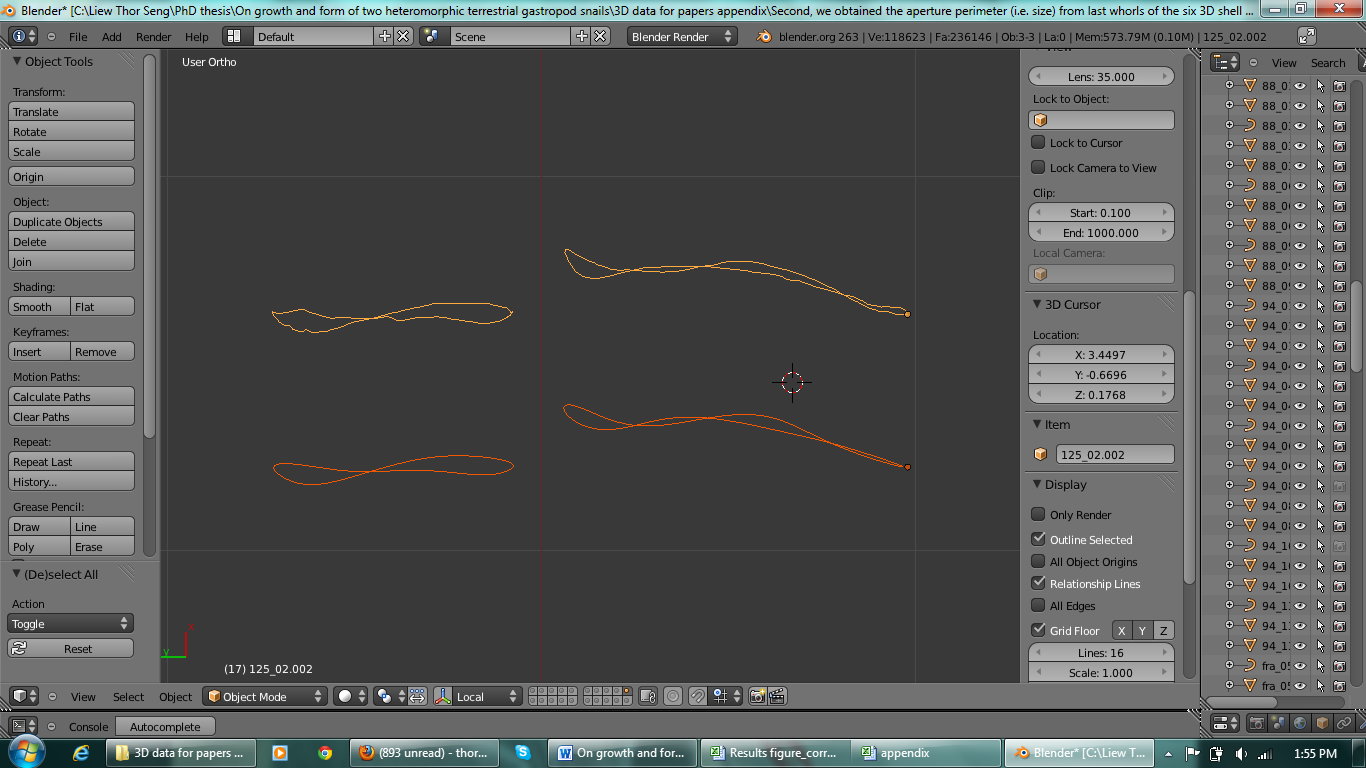


**B**

**A and B represents two different views of the same outlines. Yellow color outlines represent original aperture outlines digitized from shells. Orange color outlines represent aperture outlines that reconstruction of Elliptical Fourier analysis with 5 harmonics.**
