# Supplementary material for: On growth and form of irregular coiled-shell of a terrestrial snail: Plectostoma concinnum (Fulton, 1901) (Mollusca: Caenogastropoda: Diplommatinidae)
Source: PeerJ. 2014 May 15;2:e383. doi: 10.7717/peerj.383 (PMC4034611; doi:10.7717/peerj.383)
Supplement: File S11 [file peerj-02-383-s011.docx]

**Table 1. The proportion of variance for each principal component (PC) from the PC analysis of aperture shapes EFA descriptors.**

|  | PC1 | PC2 | PC3 | PC4 | PC5 | PC6 | PC7 | PC8 | PC9 | PC10 |
| --- | --- | --- | --- | --- | --- | --- | --- | --- | --- | --- |
| Standard deviation | 0.050 | 0.025 | 0.021 | 0.019 | 0.013 | 0.012 | 0.008 | 0.007 | 0.007 | 0.006 |
| Proportion of variance | 0.538 | 0.142 | 0.097 | 0.083 | 0.038 | 0.034 | 0.014 | 0.011 | 0.011 | 0.009 |
| Cumulative proportion | **0.538** | **0.681** | **0.777** | 0.860 | 0.898 | 0.931 | 0.946 | 0.957 | 0.968 | 0.977 |

**Table 2. Correlation between each EFA descriptor and three major principal components.**

|  | PC1 | PC2 | PC3 |
| --- | --- | --- | --- |
| A1 | -0.96** | 0.18 | -0.16 |
| B1 | -0.86** | 0.04 | 0.26 |
| C1 | -0.73** | 0.28 | 0.45* |
| D1 | -0.84** | 0.16 | -0.35 |
| E1 | -0.55** | 0.07 | 0.46* |
| F1 | 0.17 | -0.16 | 0.16 |
| A2 | 0.06 | -0.1 | 0.42* |
| B2 | 0.29 | 0.61** | 0.3 |
| C2 | -0.18 | -0.61** | -0.25 |
| D2 | 0.47* | 0.27 | 0.07 |
| E2 | 0.11 | 0.29 | -0.11 |
| F2 | -0.1 | -0.18 | 0.65** |
| A3 | 0.32 | 0.25 | 0.01 |
| B3 | -0.39* | -0.42* | -0.49** |
| C3 | -0.47* | -0.4* | 0.32 |
| D3 | 0.96** | -0.16 | 0.15 |
| E3 | 0.93** | -0.17 | 0.06 |
| F3 | -0.03 | -0.13 | -0.68** |
| A4 | -0.1 | -0.11 | 0.73** |
| B4 | -0.43* | 0.58** | 0.52** |
| C4 | -0.31 | -0.07 | 0.27 |
| D4 | -0.76** | -0.16 | 0.37* |
| E4 | -0.5** | 0 | -0.44* |
| F4 | -0.14 | 0.19 | 0.58** |
| A5 | 0.2 | 0.02 | 0.35* |
| B5 | 0.07 | 0.16 | -0.06 |
| C5 | 0.36* | 0.87** | 0.14 |
| D5 | -0.56** | -0.54** | 0.42* |
| E5 | 0.06 | 0.6** | -0.18 |
| F5 | 0.25 | -0.21 | 0.12 |

*significant at 0.05

** significant at 0.01
